# Personals

**Published:** 1913-04

**Authors:** 


### PERSONALS.

Dr. William J. Robinson was honored by a banquet, at which over 200 gathered, on March 7. The occasion was the tenth anniversary of the *Critic and Guide*. Dr. Jacobi, president of the A. M. A., presided. Various speakers, medical and otherwise, testified to Dr. Robinson's campaign against unethical products and for eugenics and better social conditions. In response, Dr. Robinson expressed especial gratification at the presence and good will of many who differed with him as to certain opinions.

Dr. Otto R. Eichel has resigned the position of superintendent of the J. N. Adam Memorial Hospital at Perrysburg and has been succeeded by Dr. Clarence L. Hyde of Buffalo, whose work in various anti-tuberculous movements, is well known. Dr. Horace L. Grasso, formerly of Buffalo, was eligible to the appointment by promotion, but preferred to confine himself to strictly medical service and not to assume executive duties.

Dr. E. M. Manwaren, of Buffalo, has recently undergone an operation.

Dr. Ludwig Aschoff, Professor of Pathology in the University of Freiburg, was the guest of the Buffalo Club on March 16, to meet the Academy of Medicine. Dr. Aschoff spoke fluently in English, without notes, describing his researches on trombosis. He has promised our readers an abstract of his lecture. A supper was served.

Dr. Allan N. Moore of Lockport fractured his right ankle on March 3, slipping on an icy step.

Dr. E. G. Mease of Dunkirk returned from a southern trip early in March.

Dr. Leonard T. Walsh and Miss Elleen Lynch of Dunkirk were married in Buffalo March 3. They will reside in Denver.

Dr. Ulysses B. Stein of Buffalo sailed March 15 for Europe.

Dr. F. M. Boyle of Buffalo returned from Washington March 11.

Dr. Joseph Brennan, who has been in New York for over three years, has returned to his former home in Buffalo.

Dr. Duncan A. Carmichael, Senior Surgeon, U. S. Public Health Service, who has been in charge of the Buffalo station for seven years, has been ordered to Vineyard Haven, Mass.

---

Dr. E. M. Tracy of Lackawanna spent a week in New York, early in March.

---

Dr. Frederick Frisch of Atlantic City, formerly of Buffalo, sailed to Europe March 8.

---

Dr. C. W. Hennington of Rochester, our Associate Editor, has been seriously ill with influenza, the only definite lesion being an amygdalitis, but is now convalescent.

---

Dr. John T. Harris of Tonawanda has been appointed Postmaster.

---

Dr. Allen A. Jones of Buffalo spent part of March in Atlantic City.

---

Dr. H. H. Wing, Professor of Animal Husbandry at Cornell, and Dr. John R. Williams of Rochester, addressed the University of Buffalo in a symposium on the Increased Cost of Living. March 22; the former speaking on the cost of production and the latter on the waste in delivery under present methods.

